# Prognostic Significance of Preprocedural N-Terminal Pro-B-Type Natriuretic Peptide Assessment in Diabetic Patients With Multivessel Coronary Disease Undergoing Revascularization

**DOI:** 10.3389/fcvm.2021.721260

**Published:** 2021-10-06

**Authors:** Le Wang, Hong-liang Cong, Jing-xia Zhang, Yue-cheng Hu, Xi-ming Li, Ying-yi Zhang, Lin Wang, Hua Yang, Li-bin Ren, Wei Qi, Chun-wei Liu

**Affiliations:** Department of Cardiology, Tianjin Chest Hospital, Tianjin, China

**Keywords:** N-terminal Pro-B-type natriuretic peptide, death, cardiovascular events, diabetes, revascularization

## Abstract

**Background and Aims:** The N-terminal pro-B-type natriuretic peptide (NT-proBNP) may predict adverse cardiovascular outcomes in patients with diabetes. However, its prognostic value in patients with multivessel disease (MVD) undergoing coronary revascularization remains unclear. This study aimed to evaluate the prognostic significance of preprocedural NT-proBNP levels in diabetic patients with MVD undergoing coronary revascularization.

**Methods:** A total of 886 consecutive diabetic patients with MVD who underwent coronary revascularization were enrolled in this study. Patients were divided into quartiles according to their pre-procedural NT-proBNP levels. Kaplan-Meier curves and Cox regression analyses were performed to evaluate the risk of cardiovascular events, including all-cause death, cardiovascular death, myocardial infarction (MI), stroke, and major adverse cardiovascular events (MACE), according to the NT-proBNP quartiles.

**Results:** During a median follow-up period of 4.2 years, 111 patients died (with 82 being caused by cardiovascular disease), 133 had MI, 55 suffered from stroke, and 250 experienced MACE. Kaplan-Meier curves demonstrated that NT-proBNP levels were significantly associated with higher incidences of all-cause death, cardiovascular death, MI, and MACE (log-rank test, *P* < 0.001, respectively). Multivariate Cox regression analysis revealed that NT-proBNP level was an independent predictor of adverse outcomes, including all-cause death (HR, 1.968; 95% CI, 1.377–2.812; *P* < 0.001), cardiovascular death (HR, 1.940; 95% CI, 1.278–2.945; *P* = 0.002), MI (HR, 1.722; 95% CI, 1.247–2.380; *P* = 0.001), and MACE (HR, 1.356; 95% CI, 1.066–1.725; *P* = 0.013). The role of NT-proBNP in predicting adverse outcomes was similar in patients with stable angina pectoris and acute coronary syndrome. Moreover, preprocedural NT-proBNP alone discriminated against the SYNTAX II score for predicting all-cause death [area under the curve (AUC), 0.662 vs. 0.626, *P* = 0.269], cardiovascular death (AUC, 0.680 vs. 0.622, *P* = 0.130), MI (AUC, 0.641 vs. 0.579, *P* = 0.050), and MACE (AUC, 0.593 vs. 0.559, *P* = 0.171). The addition of NT-proBNP to the SYNTAX II score showed a significant net reclassification improvement, integrated discrimination improvement, and improved C-statistic (all *P* < 0.05).

**Conclusion:** NT-proBNP levels were an independent prognostic marker for adverse outcomes in diabetic patients with MVD undergoing coronary revascularization, suggesting that preprocedural NT-proBNP measurement might help in the risk stratification of high-risk patients.

## Introduction

Patients with diabetes often have an advanced coronary atheroma burden, accelerated atherosclerosis, and multivessel involvement (1). Coronary artery bypass graft (CABG) and percutaneous coronary intervention (PCI) are currently the recommended revascularization options for patients with diabetes and multivessel disease (MVD) (2). Compared with optimal medical therapy alone, coronary revascularization has been shown to improve prognosis in patients with diabetes and MVD (3). Despite improvements in the treatment measures, the risk of recurrent cardiovascular events after revascularization in patients with diabetes is significantly higher than that in non-diabetic patients (4). It is well-demonstrated that MVD confers a higher risk of mortality compared with single-vessel disease (5). Therefore, the discovery of a rapidly available and reliable biomarker that identifies high-risk patients for an adverse event is of great clinical significance for improving the prognosis of diabetic patients with MVD after revascularization.

The N-terminal pro-B-type natriuretic peptide (NT-proBNP), an inactive fragment of the B-type natriuretic peptide, is a well-established biomarker for the diagnosis, prognosis, and management of heart failure (6). It has been demonstrated that elevated NT-proBNP levels are associated with a high risk of death and cardiovascular events in patients with diabetes (7–16). Moreover, NT-proBNP has also been regarded as one of the most attractive cardiovascular biomarkers for risk stratification in diabetic patients regardless of the presence of cardiovascular disease. Recent evidence suggests that the single NT-proBNP performs better than the risk model recommended by the European Society of Cardiology/European Association for the Study of Diabetes for predicting death and cardiovascular risk in patients with diabetes (17). However, the recent guidelines did not recommend routine NT-proBNP measurement for cardiovascular risk prediction in patients with diabetes (18). Although a few studies have evaluated the prognostic value of NT-proBNP in diabetic patients with coronary artery disease (CAD), the enrolled patients were restricted to patients with acute coronary syndrome (ACS) (10, 11, 19) or stable angina pectoris (SAP) (16) but not those with MVD. To date, there is a lack of insight into the prognostic significance of coronary revascularization in diabetic patients with MVD. Therefore, this study aimed to investigate the association between NT-proBNP levels and adverse outcomes, including all-cause death, cardiovascular death, myocardial infarction (MI), stroke, and major adverse cardiovascular events (MACEs) in diabetic patients with MVD undergoing coronary revascularization.

## Methods

### Study Population

This was a single-center, retrospective, observational cohort study conducted at Tianjin Chest Hospital between January 2016 and December 2016. A total of 1,629 patients with type 2 diabetes who underwent either PCI with DES or CABG surgery (elective or urgent procedure) for MVD during this hospitalization were eligible for enrollment. Patients with type 2 diabetes were defined as those with a history of type 2 diabetes who were currently receiving insulin or hypoglycemic medications. MVD was defined as severe stenosis (≥50% diameter stenosis) in at least two major epicardial coronary arteries. The following patients were excluded from the study: (1) patients with a history of PCI (*n* = 314); (2) patients with a history of CABG (*n* = 102); (3) patients with a severe valvular disease or congenital heart disease requiring cardiac surgery (*n* = 42); (4) patients lacking complete coronary artery angiography data (*n* = 38); (5) patients lacking NT-proBNP data before PCI or CABG procedure (*n* = 170); and (6) patients lacking complete clinical follow-up data (*n* = 68). Patients were followed up from January 2020 to December 2020 by telephone or outpatient clinic visits. Finally, 886 patients were included in the study. Patients were divided into quartiles according to the NT-proBNP levels before revascularization: quartile 1 (*n* = 221, NT-proBNP ≤99.30), quartile 2 (*n* = 222, 99.30< NT-proBNP ≤278.0), quartile 3 (*n* = 221, 228.0< NT-proBNP ≤1008.0), and quartile 4 (*n* = 222, NT-proBNP >1008.0). The study was approved by the local research ethics committee and complied with the Declaration of Helsinki. Given the retrospective nature of this study, no informed consent was obtained.

### Data Collection and Definitions

All clinical data were collected by two trained clinicians who were blinded to the purpose of the study. Data collected from medical records included age, sex, height, weight, duration of diabetes, smoking history, history of hypertension, history of hyperlipidemia, history of MI, history of stroke, history of chronic obstructive pulmonary disease, history of peripheral artery disease, left ventricular ejection fraction (LVEF), clinical presentation, revascularization, and medication at discharge. The following laboratory data were collected at fasting status before the procedure: fasting plasma glucose (FPG), hemoglobin A1c (HbA1c), total cholesterol, triglycerides (TG), low-density lipoprotein-C, high-density lipoprotein-C (HDL-C), serum creatinine at admission, and NT-proBNP. The estimated glomerular filtration rate was calculated according to the Modification of Diet in Renal Disease equation. Body mass index was defined as weight (kg)/height (m^2^). The severity of coronary artery lesions was calculated according to the Synergy between PCI with Taxus and Cardiac Surgery (SYNTAX) score using an online calculator (https://m.medsci.cn/scale/show.do?id=f1f22922dc). The SYNTAX II score was calculated using an online calculator (https://m.medsci.cn/scale/show.do?id=8f9d2552a8) based on two anatomical variables and six clinical variables.

### Endpoints

The primary endpoint was all-cause death. The secondary endpoints included cardiovascular death, MI, stroke, and MACEs. The causes of death were mainly determined by medical records, death certificates, and autopsy reports. All-cause death was defined as death from any cause. Cardiovascular death was defined as death due to acute MI, heart failure, cardiac arrhythmia, or stroke. MACE was defined as a composite of all-cause death, MI, and stroke.

### Statistical Analysis

Continuous variables are presented as mean ± standard deviation when normally distributed or as medians with interquartile ranges for results not normally distributed. These were compared with variance or the Kruskal-Wallis tests. NT-proBNP was transformed to a normally distributed variable using log (NT-proBNP). Categorical variables are presented as frequencies and percentages and compared using the chi-square test or Fisher's exact test. Survival analyses were performed using Kaplan-Meier analysis curves and compared using the log-rank test. Multivariate Cox proportional hazard regression analyses were used to determine the association between NT-proBNP (as a categorical and continuous variable) and endpoints and calculate the hazard ratios (HRs) and 95% confidence intervals (CIs). All the possible variables in Table 1 were introduced into the univariate model and then selected in the multivariate model when the *P*-value was <0.10. The variables included age, hyperlipidemia, previous MI, LVEF, FPG, HbA1c, HDL-C, SYNTAX score, revascularization, aspirin, and clopidogrel/ticagrelor use. Subgroup analyses for adverse outcomes were conducted according to clinical presentation (SAP and ACS). Patients were divided into low and high NT-proBNP groups according to the median value of each subgroup. Receiver-operating characteristic (ROC) curves were used to determine the predictive value of NT-proBNP for endpoints (all-cause death, cardiovascular death, MI, and MACE). Differences between the area under the ROC curve (AUC) of log (NT-proBNP) and SYNTAX II scores were compared using Hanley's test. To evaluate the discriminatory and reclassification ability of NT-proBNP beyond the SYNTAX II score for predicting endpoints, the C-statistics, category-free net reclassification improvement (NRI), and integrated discrimination improvement (IDI) were compared between the models. A two-sided analysis with a *P*-value < 0.05 was considered significant. All analyses were performed using SPSS version 20.0 (IBM Corp, Armonk, NY, USA) and SAS (version 9.1.3; Cary, NC, USA).

**Table 1:** Baseline characteristics of the study patients grouped by the NT-proBNP level.

**Clinical characteristics**	**Q1 *N* = 221**	**Q2 *N* = 222**	**Q3 *N* = 221**	**Q4 *N* = 222**	***P*** **value**
Age, years	64.3 ± 6.0	66.5 ± 6.6	66.9 ± 7.3	69.1 ± 7.4	<0.001
Male	138 (62.4)	122 (55.0)	139 (62.9)	120 (54.1)	0.105
BMI, kg/m2	25.9 ± 2.9	26.7 ± 3.0	26.9 ± 3.3	26.2 ± 3.0	0.002
Duration of diabetes, years	8.9 ± 6.8	10.1 ± 7.6	10.2 ± 7.8	11.1 ± 8.0	0.027
Smoker	95 (43.0)	94 (42.3)	106 (48.0)	85 (38.3)	0.234
Hypertension	160 (72.4)	175 (78.8)	183 (82.8)	187 (84.2)	0.009
Hyperlipidemia	141 (63.8)	141 (63.5)	144 (65.2)	151 (68.0)	0.743
Previous MI	11 (5.0)	18 (8.1)	24 (10.9)	35 (15.8)	0.001
Previous stroke	35 (15.8)	43 (19.4)	66 (29.9)	77 (34.7)	<0.001
COPD	3 (1.4)	1 (0.5)	2 (0.9)	2 (0.9)	0.786
PAD	2 (0.9)	2 (0.9)	3 (1.4)	7 (3.2)	0.225
LVEF	61 ± 5	59 ± 6	56 ± 7	48 ± 9	<0.001
Clinical presentation					<0.001
SAP	131 (59.3)	86 (38.7)	42 (19.0)	21 (9.5)	
UAP	70 (31.7)	106 (47.7)	107 (48.4)	97 (43.7)	
NSTEMI	14 (6.3)	18 (8.1)	55 (24.9)	71 (32.0)	
STEMI	6 (2.7)	12 (5.5)	17 (7.7)	33 (14.8)	
**Laboratory findings**					
FPG, mmol/L	8.1 ± 3.0	8.4 ± 3.3	8.5 ± 3.9	8.7 ± 3.4	0.406
HbA1c, %	7.5 ± 1.3	7.6 ± 1.5	7.5 ± 1.3	7.6 ± 1.5	0.582
TC, mmol/L	4.50 ± 1.21	4.47 ± 1.10	4.38 ± 1.09	4.42 ± 1.16	0.653
TG, mmol/L	1.62 (1.21–2.30)	1.59 (1.14–2.14)	1.53 (1.12–2.05)	1.46 (1.11–1.99)	0.047
LDL-C, mmol/L	2.96 ± 1.08	2.93 ± 0.94	2.92 ± 0.96	2.92 ± 0.96	0.973
HDL-C, mmol/L	1.05 ± 0.28	1.02 ± 0.28	1.00 ± 0.27	1.00 ± 0.26	0.308
NT-proBNP, pg/ml	58.9 (39.8–78.9)	157.3 (121.7–211.7)	548.3 (377.9–812.0)	2208.5 (1500.0–3664.5)	<0.001
eGFR, mL/min	89 ± 21	91 ± 20	89 ± 24	79 ± 23	<0.001
Left main disease	29 (13.1)	31 (14.0)	32 (14.5)	39 (17.6)	0.576
SYNTAX score	19.9 ± 8.6	22.3 ± 8.4	23.1 ± 9.0	24.7 ± 9.7	<0.001
SYNTAX II score	26.8 ± 5.4	28.4 ± 6.1	29.7 ± 6.7	34.6 ± 8.8	<0.001
Revascularization					0.104
PCI	184 (83.3)	170 (76.6)	182 (82.4)	168 (75.7)	
CABG	37 (16.7)	52 (23.4)	39 (17.6)	54 (24.3)	0.067
Complete revascularization	102 (46.2)	92 (41.4)	75 (33.9)	94 (42.3)	
**Medications at discharge**					
Aspirin	220 (99.5)	220 (99.1)	221 (100.0)	222 (100.0)	0.109
Clopidogrel/Ticagrelor	219 (99.1)	219 (98.6)	220 (99.5)	216 (97.3)	0.209
β-blocker	144 (65.2)	151 (68.0)	175 (79.2)	176 (79.3)	<0.001
ACEI/ARB	119 (53.8)	124 (55.9)	154 (69.7)	155 (69.8)	<0.001
Statin	212 (95.9)	210 (94.6)	218 (98.6)	215 (96.8)	0.128
Insulin	70 (31.7)	99 (44.6)	86 (38.9)	95 (42.8)	0.029
Metformin	59 (26.7)	56 (25.2)	52 (23.5)	59 (26.6)	0.858
Sulphonyurea	39 (17.6)	34 (15.3)	34 (15.4)	30 (13.5)	0.692
Non-sulfonylurea insulin	35 (15.8)	28 (12.6)	31 (14.0)	25 (11.3)	0.533
Secretagogue	116 (52.5)	109 (49.1)	121 (54.8)	127 (57.2)	0.365
Glucosidase inhibitors other OADs	9 (4.1)	7 (3.2)	8 (3.6)	5 (2.3)	0.736

*Data are expressed as mean ± SD, medians with interquartile ranges or percentage. NT-proBNP, N-terminal proB-type natriuretic peptide; BMI, body mass index; MI, myocardial infarction; COPD, chronic obstructive pulmonary disease; PAD, peripheral artery disease; LVEF, left ventricle ejection fraction; SAP, stable angina pectoris; UAP, unstable angina pectoris; NSTEMI, non-ST-segment elevation myocardial infarction; STEMI, ST-segment elevation myocardial infarction; FBG, fasting plasm glucose; HbA1c, Hemoglobin A1c; TC, total cholesterol; TG, triglycerides; LDL-C, low-density lipoprotein cholesterol; HDL-C, high-density lipoprotein cholesterol; eGFR, estimated glomerular filtration rate; PCI, percutaneous coronary intervention; CABG, coronary artery bypass graft; ACEI, angiotensin II coenzyme inhibitor; ARB, angiotensin II receptor blocker; OADs, oral antidiabetic drugs*.

## Results

### Baseline Characteristics

Of the 886 patients in the present study, 58.6% were men, and the mean age was 66.7 ± 7.0 years. The median NT-proBNP level of all the patients was 279.9 pmol/L (interquartile range: 99.4–1010.0 pmol/L). The baseline clinical, laboratory, angiographic, and treatment characteristics of the patients according to NT-proBNP quartiles are listed in Table 1. The median NT-proBNP levels of the four groups were 58.9 pg/ml, 157.3 pg/ml, 548.3 pg/ml, and 2208.5 pg/ml, respectively.

There were significant differences among the four groups in terms of age, body mass index, duration of diabetes, prevalence of hypertension, previous MI, previous stroke, LVEF, clinical presentation, triglycerides, estimated glomerular filtration rate, SYNTAX score, SYNTAX II score, and use of β-blocker, ACE inhibitor/angiotensin receptor blocker, and insulin (all *P* < 0.05). There were no significant differences in other variables among the four groups.

Patients with SAP were divided into low and high NT-proBNP groups according to the median value of NT-proBNP levels (110.6 pg/ml). The median NT-proBNP level of the low and high NT-proBNP groups were 62.9 pg/ml (interquartile range: 39.9–82.9 pg/ml) and 260.0 pg/ml (interquartile range: 163.0–566.9 pg/ml). Patients with ACS were divided into low and high NT-proBNP groups according to the median value of NT-proBNP levels (527.4 pg/ml). The median NT-proBNP level of the low and high NT-proBNP groups were 149.3 pg/ml (interquartile range: 83.2–289.6 pg/ml) and 1532.0 pg/ml (interquartile range: 918.3–2686.0 pg/ml). In the group with low NT-proBNP level, 224 (73.9%) patients had unstable angina pectoris (UAP), while 53(17.5%) patients had non-ST-segment elevation myocardial infarction (NSTEMI), and 26(8.6%) patients had ST-segment elevation myocardial infarction (STEMI). In the group with high NT-proBNP level, 156 (51.5%) patients had UAP, while 105 (34.7%) patients had NSTEMI, and 42 (13.8%) patients had STEMI. There was significant difference in the distribution of ACS presentation in the groups with low and high NT-proBNP levels (*P* < 0.001).

### Cardiovascular Events During Follow-up

During a median 4.2-year follow-up period, 250 (28.2%) patients experienced MACE, which comprised 111 (12.5%) all-cause deaths, 133 (15.0%) MI, and 55 (6.2%) strokes. Of the 111 all-cause deaths, 82 (73.9%) were caused by cardiovascular disease. For SAP group, 72 (25.7%) patients experienced MACE, which comprised 28 (10.0%) all-cause deaths, 31 (11.1%) MI, and 21 (7.5%) strokes. Of the 28 all-cause deaths, 21 (75.0%) were caused by cardiovascular disease. For ACS group, 178 (29.4%) patients experienced MACE, which comprised 83 (13.7%) all-cause deaths, 102 (16.8%) MI, and 34 (5.6%) strokes. Of the 83 all-cause deaths, 60 (72.3%) were caused by cardiovascular disease. All-cause mortality across UAP group, NSTEMI group and STEMI group was 11.6, 17.1, 17.6%, respectively (*P* = 0.144). Cardiovascular mortality across UAP group, NSTEMI group and STEMI group was 7.9, 13.3, 13.2%, respectively (*P* = 0.100).

The incidence of MI across UAP group, NSTEMI group and STEMI group was 14.5, 21.5, 19.1%, respectively (*P* = 0.120). The incidence of stroke across UAP group, NSTEMI group and STEMI group was 4.2, 9.5, 4.4%, respectively (*P* = 0.064). The incidence of MACE across UAP group, NSTEMI group and STEMI group was 26.8, 32.9, 35.3%, respectively (*P* = 0.194).

### Predictive Role of Preprocedural NT-proBNP

**Figure 1:**
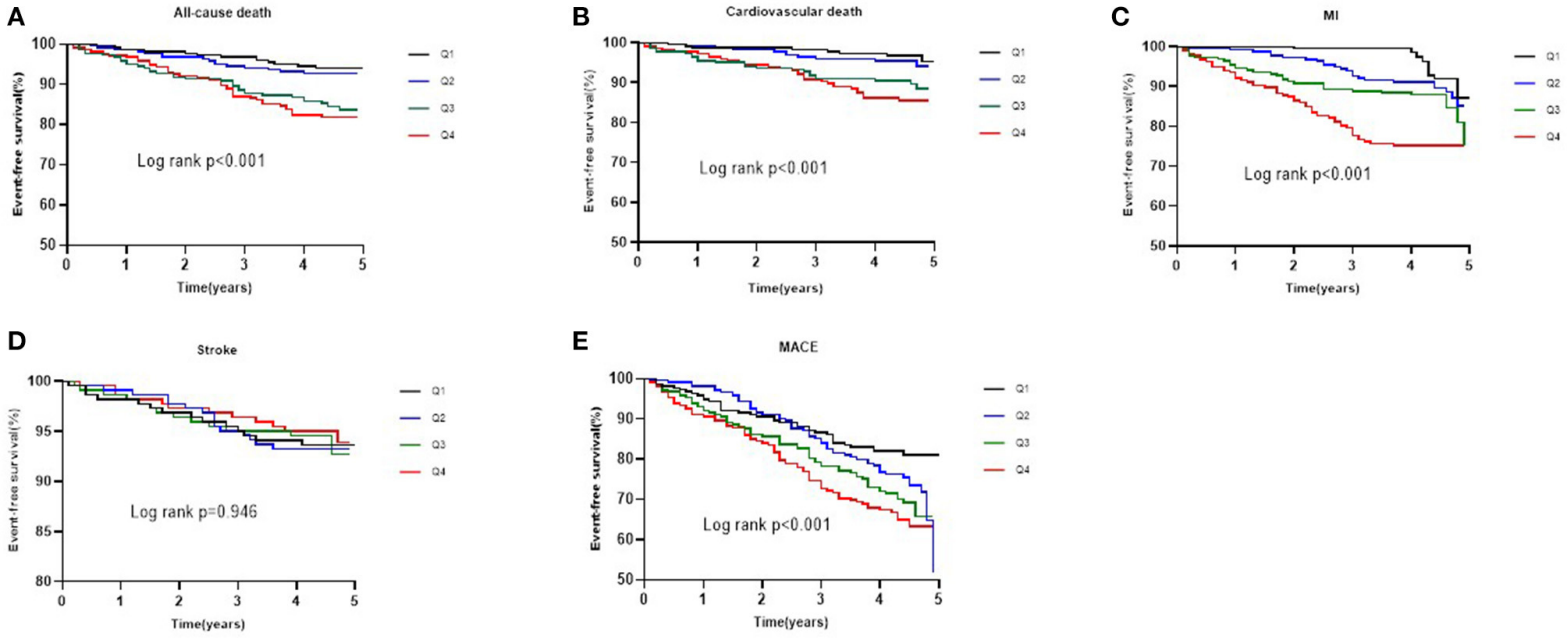
Kaplan-Meier survival curve for all-cause death **(A)**, cardiovascular death **(B)**, MI **(C)**, stroke **(D)**, and MACE **(E)** across NT-proBNP quartiles.

As shown in Figure 1, the Kaplan-Meier survival analysis showed that the cumulative incidence of all-cause death, cardiovascular death, MI, and MACE increased with higher quartiles of NT-proBNP levels (log-rank test, *P* < 0.001). However, there was no significant difference in the incidence of stroke among the four groups (log-rank test, *P* = 0.946).

**Figure 2:**
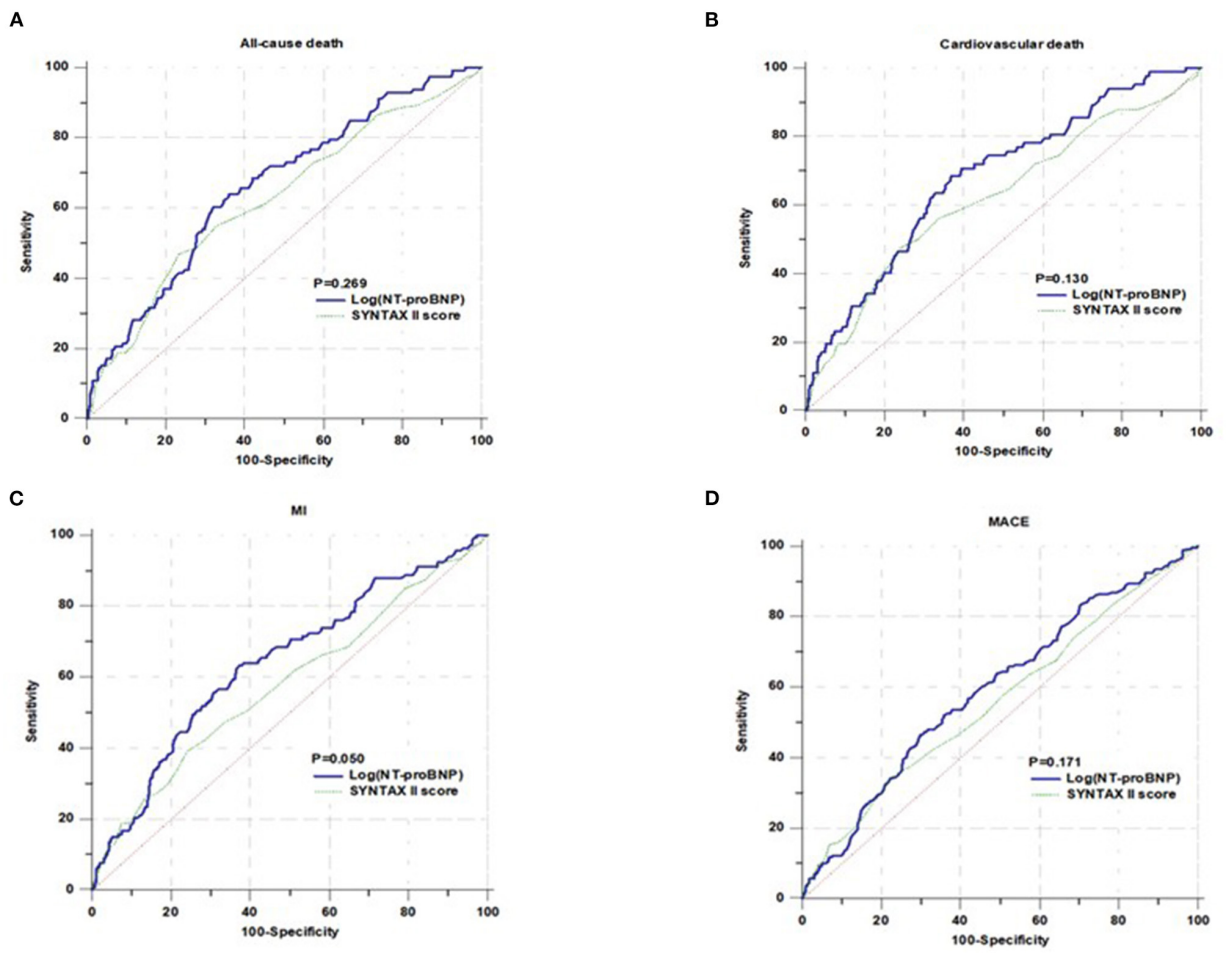
Comparison of area under the receiver operating characteristic cures for predicting all-cause death **(A)**, cardiovascular death **(B)**, MI **(C)**, and MACE **(D)**.

As shown in Table 2, in the univariate analysis, there was a direct correlation between NT-proBNP levels and the rate of all-cause death, cardiovascular death, MI, and MACE, but not stroke. The association persisted even after the adjustments for age, hyperlipidemia, previous MI, LVEF, FPG, HbA1c, HDL-C, SYNTAX score, revascularization, and aspirin and clopidogrel/ticagrelor use. When considered as a categorical variable, more pronounced HRs were found between NT-proBNP and all-cause death (quartile 4 vs. quartile 1, adjusted HR 2.648, 95% CI: 1.254–5.594, *P* = 0.011), cardiovascular death (quartile 4 vs. quartile 1, adjusted HR 3.346, 95% CI: 1.314–8.520, *P* = 0.011), MI (quartile 4 vs. quartile 1, adjusted HR 3.038, 95% CI: 1.589–5.808, *P* = 0.001), and MACE (quartile 4 vs. quartile 1, adjusted HR 1.828, 95% CI: 1.155–2.895, *P* = 0.010). When regarded as a continuous variable per unit increase in log-transformed NT-proBNP levels, multivariate Cox regression analysis showed that NT-proBNP was independently associated with all-cause death (adjusted HR 1.968, 95% CI: 1.377–2.812, *P* < 0.001), cardiovascular death (adjusted HR 1.940, 95% CI:1.278–2.945, *P* = 0.002), MI (adjusted HR 1.722, 95% CI: 1.247–2.380, *P* = 0.001), and MACE (adjusted HR 1.356, 95% CI: 1.066–1.725, *P* = 0.013). As shown in Table 3, the relationship of NT-proBNP (high or low) with adverse outcomes was consistent in patients with SAP and ACS. After adjustment for covariates, NT-proBNP remained an independent predictor for all-cause death, cardiovascular death, MI and MACE in patients with SAP and ACS (all *P* < 0.05). As shown in Table 4, the ROC analysis showed that the AUC was 0.662 (95% CI: 0.630–0.694, *P* < 0.001) for all-cause death, 0.680 (95% CI: 0.648–0.711, *P* < 0.001) for cardiovascular death, 0.641 (95% CI: 0.608–0.672, *P* < 0.001) for MI, and 0.593 (95% CI: 0.560–0.625, *P* < 0.001) for MACE. As shown in Figure 2, ROC curve analysis showed that there was no significant difference in the SYNTAX II score model compared with NT-proBNP for predicting all-cause death (*P* = 0.269), cardiovascular death (*P* = 0.130), MI (*P* = 0.050), and MACE (*P* = 0.171).

**Table 2:** Cox univariate and multivariate analysis of the association between NT-proBNP levels and endpoints.

**Outcomes**	**Events, *n*/Total**	**Crude HR (95% CI)**	***P-value***	**Adjusted HR (95% CI)**	***P-value***
**All-cause death**					
Q1	13/221	Reference		Reference	
Q2	18/222	1.400 (0.686–2.858)	0.355	1.264 (0.598–2.672)	0.540
Q3	35/221	2.842 (1.504–5.371)	0.001	2.697 (1.350–5.388)	0.005
Q4	45/222	3.740 (2.017–6.933)	<0.001	2.648 (1.254–5.594)	0.011
Log (NT–proBNP) per 1 log unit		2.315 (1.753–3.057)	<0.001	1.968 (1.377–2.812)	<0.001
**Cardiovascular death**					
Q1	8/221	Reference		Reference	
Q2	13/222	1.643 (0.681–3.964)	0.269	1.539 (0.600–3.945)	0.369
Q3	24/221	3.119 (1.401–6.942)	0.005	3.110 (1.285–7.528)	0.012
Q4	37/222	4.925 (2.294–10.577)	<0.001	3.346 (1.314–8.520)	0.011
Log (NT-proBNP) per 1 log unit		2.552 (1.846–3.530)	<0.001	1.940 (1.278–2.945)	0.002
**MI**					
Q1	16/221	Reference		Reference	
Q2	26/222	1.607 (0.862–2.996)	0.135	1.264 (0.666–2.397)	0.474
Q3	33/221	2.168 (1.193–3.938)	0.011	1.956 (1.053–3.636)	0.034
Q4	58/222	3.810 (2.190–6.627)	<0.001	3.038 (1.589–5.808)	0.001
Log (NT-proBNP) per 1 log unit		2.106 (1.632–2.716)	<0.001	1.722 (1.247–2.380)	0.001
**Stroke**					
Q1	14/221	Reference		Reference	
Q2	15/222	1.055 (0.509–2.185)	0.886	1.143 (0.534–2.445)	0.731
Q3	14/221	0.991 (0.472–2.078)	0.981	1.097 (0.492–2.445)	0.821
Q4	12/222	0.840 (0.389–1.817)	0.658	0.996 (0.380–2.609)	0.993
Log (NT-proBNP) per 1 log unit		0.869 (0.579–1.306)	0.500	0.892 (0.527–1.508)	0.669
**MACE**					
Q1	40/221	Reference		Reference	
Q2	59/222	1.506 (1.005–2.258)	0.047	1.300 (0.855–1.976)	0.220
Q3	68/221	1.847 (1.246–2.738)	0.002	1.698 (1.119–2.574)	0.013
Q4	83/222	2.237 (1.529–3.274)	<0.001	1.828 (1.155–2.895)	0.010
Log (NT-proBNP) per 1 log unit		1.499 (1.244–1.805)	<0.001	1.356 (1.066–1.725)	0.013

*Adjusted variables were age, hyperlipidemia, previous MI, LVEF, FPG, HbA1c, HDL-C, SYNTAX score, revascularization, aspirin and clopidogrel/ticagrelor use. NT-proBNP, N-terminal proB-type natriuretic peptide; MI, myocardial infarction; MACE, major adverse cardiovascular event; HR, hazard ratio; CI, confidential interval*.

**Table 3:** Subgroup analysis of the association between NT-proBNP levels and endpoints according to clinical presentation.

	**SAP**					**ACS**				
**Outcomes**	**Events, *n*/Total**	**Crude HR (95% CI)**	***P-value***	**Adjusted HR (95% CI)**	***P-value***	**Events, *n*/Total**	**Crude HR (95% CI)**	***P-value***	**Adjusted HR (95% CI)**	***P-value***
**All-cause death**										
Low NT-proBNP	8/140	Reference		Reference		21/303	Reference		Reference	
High NT-proBNP	20/140	2.653 (1.168–6.024)	0.020	2.669 (1.085–6.564)	0.032	62/303	3.190 (1.945–5.233)	<0.001	2.854 (1.636–4.977)	<0.001
Log (NT–proBNP)		2.617 (1.395–4.909)	0.003	2.849 (1.309–6.200)	0.008		2.442 (1.724–3.457)	<0.001	1.907 (1.234–2.947)	0.004
**Cardiovascular death**										
Low NT-proBNP	5/140	Reference		Reference		13/303	Reference		Reference	
High NT-proBNP	17/140	3.636 (1.341–9.859)	0.011	3.492 (1.122–10.866)	0.031	47/303	3.828 (2.071–7.076)	<0.001	3.171 (1.598–6.289)	0.001
Log (NT-proBNP)		3.507 (1.763–6.978)	<0.001	3.362 (1.375–8.220)	0.008		2.723 (1.804–4.111)	<0.001	1.843 (1.103–3.081)	0.020
**MI**										
Low NT-proBNP	5/140	Reference		Reference		30/303	Reference		Reference	
High NT–proBNP	26/140	5.862 (2.248–15.285)	<0.001	5.805 (2.142–15.733)	0.001	72/303	2.685 (1.754–4.112)	<0.001	2.176 (1.339–3.538)	0.002
Log (NT-proBNP)		2.738 (1.496–5.012)	0.001	3.213 (1.535–6.722)	0.002		2.185 (1.597–2.989)	<0.001	1.500 (1.022–2.201)	0.038
**Stroke**										
Low NT-proBNP	13/140	Reference		Reference		17/303	Reference		Reference	
High NT-proBNP	8/140	0.597 (0.247–1.442)	0.252	0.585 (0.212–1.615)	0.301	17/303	1.000 (0.510–1.958)	0.999	0.994 (0.452–2.182)	0.987
Log (NT-proBNP)		0.703 (0.286–1.724)	0.441	0.651 (0.223–1.905)	0.433		1.052 (0.626–1.769)	0.847	1.047 (0.538–2.040)	0.892
**MACE**										
Low NT-proBNP	25/140	Reference		Reference		66/303	Reference		Reference	
High NT-proBNP	47/140	2.122 (1.297–3.471)	0.003	2.079 (1.216–3.554)	0.007	112/303	1.793 (1.322–2.430)	<0.001	1.651 (1.162–2.346)	0.005
Log (NT-proBNP)		1.678 (1.098–2.563)	0.017	1.806 (1.095–2.979)	0.021		1.622 (1.284–2.049)	<0.001	1.345 (1.002–1.804)	0.048

*Adjusted variables were age, hyperlipidemia, previous MI, LVEF, FPG, HbA1c, HDL-C, SYNTAX score, revascularization, aspirin and clopidogrel/ticagrelor use. NT-proBNP, N-terminal proB-type natriuretic peptide; MI, myocardial infarction; MACE, major adverse cardiovascular event; HR, hazard ratio; CI, confidential interval; SAP, stable angina pectoris; ACS, acute coronary syndrome*.

**Table 4:** Receiver operating characteristic curve evaluation of Log (NT-proBNP) for endpoints prediction.

	**Area under the curve**	**95% CI**	**Sensitivity**	**Specificity**	***P- value***
All-cause death	0.662	0.630–0.694	60.4%	68.0%	<0.001
Cardiovascular death	0.680	0.648–0.711	68.3%	63.3%	<0.001
MI	0.641	0.608–0.672	63.2%	62.8%	<0.001
MACE	0.593	0.560–0.625	46.4%	70.1%	<0.001

*MI, myocardial infarction; MACE, major adverse cardiovascular event; CI, confidential interval*.

### Incremental Value of NT-proBNP Beyond the SYNTAX II Score

As shown in Table 5, the C-statistic of the SYNTAX II score model was 0.627 (95% CI: 0.569–0.685) for all-cause death, 0.627 (95% CI: 0.569–0.685) for all-cause death, 0.622 (95% CI: 0.553–0.690) for cardiovascular death, 0.579 (95% CI: 0.523–0.634) for MI, and 0.559 (95% CI: 0.516–0.602) for MACE. Adding NT-proBNP to the SYNTAX II score model increased the C-statistic to 0.674 (95% CI: 0.620–0.728, *P* = 0.049), 0.687 (95% CI: 0.626–0.748, *P* = 0.035), 0.644 (95% CI: 0.592–0.697, *P* = 0.011), and 0.595 (95% CI: 0.553–0.637, *P* = 0.43), respectively. Moreover, the addition of NT-proBNP to the SYNTAX II score model resulted in a significant increase in the NRI and IDI (all *P* < 0.05).

**Table 5:** Prognostic information provided by NT-proBNP beyond SYNTAX II score.

	**C-index (95% CI)**	***P- value***	**NRI (95% CI)**	***P-value***	**IDI (95% CI)**	***P-value***
**All cause death**						
SYNTAX II score	0.627 (0.569–0.685)	Reference		Reference		Reference
SYNTAX II score +Log (NT-proBNP)	0.674 (0.620–0.728)	0.049	0.294 (0.097–0.491)	0.004	0.023 (0.013–0.034)	<0.001
**Cardiovascular death**						
SYNTAX II score	0.622 (0.553–0.690)	Reference		Reference		Reference
SYNTAX II score + Log (NT-proBNP)	0.687 (0.626–0.748)	0.035	0.343 (0.118–0.567)	0.003	0.024 (0.012–0.036)	<0.001
**MI**						
SYNTAX II score	0.579 (0.523–0.634)	Reference		Reference		Reference
SYNTAX II score + Log (NT-proBNP)	0.644 (0.592–0.697)	0.011	0.312 (0.129–0.494)	<0.001	0.021 (0.011–0.032)	<0.001
**MACE**						
SYNTAX II score	0.559 (0.516–0.602)	Reference		Reference		Reference
SYNTAX II score + Log (NT-proBNP)	0.595 (0.553–0.637)	0.043	0.144 (0.002–0.286)	0.018	0.012 (0.004–0.019)	0.002

*NT-proBNP, N-terminal pro-B-type natriuretic peptide; MI, myocardial infarction; MACE, major adverse cardiovascular event; NRI, net reclassification improvement; IDI, integrated discrimination*.

## Discussion

The present study evaluated the prognostic implications of pre-procedural NT-proBNP levels in diabetic patients with MVD undergoing coronary revascularization. The major findings were as follows: First, higher NT-proBNP levels were associated with an increased risk of all-cause death, cardiovascular death, MI, and MACE, but not stroke. The association between NT-proBNP levels and cardiovascular events persisted and was consistent in patients with SAP and ACS after adjustment for potential confounding factors. Second, NT-proBNP had a discriminatory ability for death and cardiovascular events, such as the SYNTAX II score. Third, adding NT-proBNP to the SYNTAX II score significantly improved the discriminatory and reclassification ability of the risk of death and cardiovascular events. Our results indicate that pre-procedural NT-proBNP measurement before coronary revascularization in diabetic patients with MVD is valuable for risk stratification. Most importantly, the present study suggested that the incorporation of NT-proBNP into SYNTAX II score risk algorithms may provide more accurate risk stratification for death and cardiovascular events. To the best of our knowledge, the present study revealed, for the first time, the prognostic utility of NT-proBNP in diabetic patients with MVD undergoing revascularization.

It is well-known that multivessel CAD is associated with worse clinical outcomes compared with single-vessel CAD. Although revascularization may improve the prognosis in patients with MVD compared with medical treatments, multivessel involvement in diabetes can impair long-term outcomes following coronary revascularization (20). Incremental cardiovascular risk is not uniformly distributed among all patients with diabetes (21). Therefore, individualized and precise risk assessment is needed in patients with diabetes and MVD who are undergoing coronary revascularization. In addition to traditional cardiovascular risk factors, multiple biomarkers have been explored to provide prognostic information in patients with diabetes and CAD (22–24). However, to the best of our knowledge, none of these studies have focused on patients with concurrent diabetes and MVD. The SYNTAX II score, which is based on the combination of two anatomical variables and six clinical variables, was developed as a tool for risk assessment and treatment guidance in patients with MVD (25). It has been demonstrated to be superior to the SYNTAX score for risk prediction. Moreover, it has a similar performance regardless of the diabetes status (26). However, the SYNTAX II score only had a modest discriminatory ability for death and cardiovascular event prediction in diabetic patients undergoing revascularization for MVD (26), suggesting that traditional risk factors do not explain the full spectrum of cardiovascular risk in patients with diabetes. Therefore, the predictive power of the current SYNTAX II score evaluation system should be strengthened. This can be done through the identification of additional biomarkers that provide incremental prognostic value beyond the SYNTAX II score.

Recently, increasing evidence has suggested that NT-proBNP, an established biomarker for the diagnosis and prognosis of heart failure, is associated with the risk of incident diabetes (27, 28) and diabetes-related microvascular and macrovascular complications (29). Moreover, higher NT-proBNP levels are associated with an increased risk of death and cardiovascular events in patients with diabetes. For type 2 diabetes mellitus patients, regardless of the presence or absence of cardiovascular disease, NT-proBNP may refine the risk prediction for death and cardiovascular events and improve the predictive powers of traditional risk models. In patients with type 2 diabetes mellitus with chronic coronary syndrome and normal left ventricular systolic function, the addition of NT-proBNP to the model of established risk factors significantly improved the risk prediction for MACE (16). For patients with type 2 diabetes mellitus after recent acute coronary syndrome, the use of NT-proBNP and clinical variables may facilitate risk stratification for expanded heart failure outcomes (19). Furthermore, a recent study demonstrated that NT-proBNP was an important predictor of poor prognosis and improved cardiovascular risk prediction beyond the SYNTAX II score in patients with a three-vessel disease (30). These findings reflect the potential prognostic utility of NT-proBNP in patients with diabetes and MVD. A preprocedural increase in NT-proBNP has been demonstrated to be associated with an increased risk of death and adverse cardiovascular events in patients undergoing coronary revascularization (31). However, it is not known whether NT-proBNP has sufficient discriminatory ability, similar to externally validated risk models in diabetic patients with MVD undergoing revascularization.

The present study extended the previous findings to a novel population of diabetic patients with CAD and showed that elevated NT-proBNP level, either as a categorical variable or as a continuous variable, was associated with an increased risk of all-cause death, cardiovascular death, MI, and MACE. Moreover, the higher risk of death and cardiovascular events persisted after a more comprehensive adjustment for potential confounding factors, including traditional risk factors, disease severity, coronary anatomy severity, laboratory markers, and treatments. Therefore, NT-proBNP level is a strong independent predictor of cardiovascular risk in diabetic patients with an advanced coronary atheroma burden. The relationship between NT-proBNP and adverse outcomes was consistent in patients with SAP and ACS.NT-proBNP predicted cardiovascular death better than the other endpoints, and it seemed to be more specific for increased cardiovascular death risk, emphasizing its clinical relevance as a specific marker of cardiovascular events in patients with diabetes. Unlike previous findings, the present study found, for the first time, that the predictive value of preprocedural NT-proBNP level for all-cause death, cardiovascular death, MI, and MACE was comparable to that of the SYNTAX II score, indicating that the former may provide the same risk prediction for death and cardiovascular events as the combination of coronary anatomy and demographic and clinical factors. More importantly, the present study further demonstrated significant improvements in the C-index, NRI, and IDI after the incorporation of NT-proBNP into the SYNTAX II score, indicating that this addition may significantly improve the discrimination and reclassification of long-term death and cardiovascular events. However, this study failed to show a significant association between NT-proBNP and the risk of stroke, indicating the need for further studies with larger sample sizes and extended follow-up times to confirm our findings.

Based on the above findings, preprocedural NT-proBNP may be amenable for use in risk stratification for diabetic patients with MVD undergoing revascularization since it is a more objective and easily available tool for predicting adverse outcomes compared with the SYNTAX II score. NT-proBNP measurement is a routine procedure for patients with CAD before angiography. Thus, the single preprocedural NT-proBNP measurement may be pragmatic in predicting death and cardiovascular events in patients with diabetes and MVD undergoing coronary revascularization. However, the current study does not suggest that NT-proBNP should replace the SYNTAX II score for risk stratification and cardiovascular evaluation. In contrast, NT-proBNP combined with the SYNTAX II score should be considered when stratifying diabetes patients with MVD before coronary revascularization. Therefore, whether adding NT-proBNP will help to build a more accurate risk score in diabetic patients with MVD needs to be further explored in future multicenter prospective studies with larger sample sizes.

The exact mechanisms underlying the association between NT-proBNP and the risk of death and cardiovascular events in patients with diabetes are not fully understood. It is known that the rise in circulating NT-proBNP levels reflects responses to increased stress on cardiomyocytes and volume overload. Although NT-proBNP is a reliable biomarker of left ventricular systolic and diastolic dysfunction (32), the association between NT-proBNP and cardiovascular risk cannot be completely attributed to the declining cardiac function. NT-proBNP concentration is positively associated with myocardial ischemia burden (33), microvascular damage (34), left ventricular hypertrophy (35), myocardial fibrosis (36), and central aortic stiffness (37). Moreover, elevated NT-proBNP levels reflect the accumulation of cardiovascular risk factors. NT-proBNP has been correlated with macro- and microvascular complications (29), such as MI, stroke, peripheral arterial disease, and diabetes-related nephropathy, retinopathy, and neuropathy, which all lead to poor cardiovascular outcomes. However, the exact mechanism in diabetic patients with MVD needs to be further elucidated.

This study had some limitations that need to be addressed. First, this was a single-center, retrospective study that may have been affected by potential selection and measurement biases. Patients undergoing primary PCI were excluded due to the lack of preprocedural NT-proBNP measurements, which may have limited the generalizability of these findings. Second, the present analyses were based on a preprocedural and single measurement. Therefore, the predictive value of dynamic measurement of NT-proBNP needs to be further evaluated. Third, the c-statistic of NT-proBNP was lower compared to the previously reported values in patients with diabetes. This discrepancy may have been due to the different burdens of comorbidities and glycemia levels. Finally, the different presentations of ACS are associated with distinct long-term risks. There was significant difference in the distribution of ACS presentation in the groups with low and high NT-proBNP levels. Patients with higher NT-proBNP levels tended to be present with more serious ACS. However, there was no significant difference in the incidence of adverse outcomes in the ACS subgroups in the present study. Further analysis was not conducted to provide data regarding the contribution of the distinct ACS presentation to adverse outcomes in the groups with low and high NT-proBNP levels due to the relatively small number of patients enrolled in the study. Therefore, future studies with larger sample size are required.

## Conclusion

The present study demonstrated for the first time that preprocedural NT-proBNP was an independent predictor of adverse outcomes in diabetic patients with MVD undergoing coronary revascularization, suggesting that routine preprocedural NT-proBNP measurement might help further risk stratification in high-risk patients.

## Data Availability Statement

The original contributions presented in the study are included in the article/supplementary material, further inquiries can be directed to the corresponding author/s.

## Ethics Statement

The studies involving human participants were reviewed and approved by the Ethical Committee of Tianjin Chest Hospital. Written informed consent for participation was not required for this study in accordance with the national legislation and the institutional requirements.

## Author Contributions

LeW, H-lC, and J-xZ participated in the study design. LeW, Y-cH, X-mL, Y-yZ, LinW, HY, L-bR, WQ, and C-wL participated in data collection. LeW, HY, and L-bR performed the statistical analysis. LeW drafted the article. All authors contributed to the article and approved the submitted version.

## Conflict of Interest

The authors declare that the research was conducted in the absence of any commercial or financial relationships that could be construed as a potential conflict of interest.

## Publisher's Note

All claims expressed in this article are solely those of the authors and do not necessarily represent those of their affiliated organizations, or those of the publisher, the editors and the reviewers. Any product that may be evaluated in this article, or claim that may be made by its manufacturer, is not guaranteed or endorsed by the publisher.
